# Impact of home exercise program on self-efficacy and quality of life among primary knee osteoarthritis patients: a randomized controlled clinical study

**DOI:** 10.1186/s43166-021-00073-2

**Published:** 2021-06-24

**Authors:** Wafaa K. Makarm, Doaa M. Sharaf, Rabab S. Zaghlol

**Affiliations:** 1 Rheumatology and Rehabilitation Department, Faculty of Medicine, Zagazig University, Zagazig, Egypt; 2 Zagazig, Egypt; 3 Nasr, Egypt; 4 Physical Medicine and Rehabilitation Department, Security Forces Hospital, Makkah, Saudi Arabia

**Keywords:** Osteoarthritis, Knee, Adherence, Home exercise, Self-efficacy, Quality of life

## Abstract

**Background:**

Knee osteoarthritis (KOA) is a common joint disorder in elderly individuals, causing pain, loss of physical functioning, disability, and reduction of life quality. Home exercise programs (HEP) serve as a crucial complement to outpatient rehabilitation therapy, as they save the cost of supervised physical therapy sessions, while also offering a high level of treatment. The aim of this study was to evaluate the effectiveness of the 6-month HEP on pain, quality of life, and self-efficacy in patients with primary KOA and to identify the adherence level to exercises and associations with patients’ characteristics and clinical outcomes.

**Results:**

After 6 months of HEP, there were statistically significant differences between groups for self-efficacy (*p* ≤ 0.001, from 58.29 to 71.5) (*p* = 0.23, from 55.98 to 57.72), quality of life (*p* ≤0.001, from 60.1 to 72.2) (*p* = 0.074, from 60.35 to 60.92), and pain severity (*P* ≤0.001, from 58.29 to 41.4) (*P* = 0.88, from 61.2 to 60.9) in favor of exercise group.

**Conclusions:**

Home-based exercise program improves pain score, self-efficacy, and quality of life in patients with knee osteoarthritis. Adherence level to the exercise program may have a positive impact on patient improvement.

**Supplementary Information:**

The online version contains supplementary material available at 10.1186/s43166-021-00073-2.

## Background

Knee osteoarthritis (KOA) is a common joint disorder in elderly individuals, causing pain, loss of physical functioning, disability, and reduction of life quality [1]. Non-pharmacological approaches, e.g., patient education, weight reduction, coping strategies, exercise, and physical therapies, are recommended for management of KOA [2].

Home exercise programs (HEP) serve as a crucial complement to outpatient rehabilitation therapy, as they save the cost of supervised physical therapy sessions, while also offering a high level of treatment [3]. This recommendation of HEP is now overemphasized during the coronavirus disease 2019 (COVID-19) pandemic which causes quarantine or lockdown; it may be a suitable and preferred choice for patients suffering from KOA who are often unable to make direct personal contact with their physician [4].

Knee pain may induce worsening of self-efficacy with deterioration of the quality of life and physical dependency [5]. Self-efficacy relates to the patient confidence level and exercise adherence. Exercise programs may attract patients with a greater belief in their abilities to manage their diseases [6].

Most previous studies have focused on the effect of home-based exercise programs on pain and quality of life measures in knee osteoarthritis [7], however, rarely considering self-efficacy.

Therefore, the present study aimed to investigate the effect of 26 weeks of HEP on pain severity, self-efficacy, and quality of life scores in primary knee osteoarthritis patients and to determine adherence to exercises in the HEP intervention group. We hypothesized that participants receiving HEP will show improvement of pain severity, self-efficacy, and life quality compared to those receiving health education without HEP.

## Methods

### Study design

A single blinded parallel randomized, controlled clinical study was conducted in accordance with CONSORT guidelines.

### Participants and setting

#### Eligibility criteria

##### Inclusion criteria

Individuals aged 40–65 years who met the American College of Rheumatology clinical and radiological criteria [8] for primary KOA were recruited from the outpatient clinic of rheumatology and rehabilitation department of university hospitals during the period of July 2018 through July 2020. The participants with knee pain for at least 3 months and of radiographic severity ≥ grade 2 according to Kellgren–Lawrence were included in the study [9]. A written informed consent was obtained before enrollment to this study.

##### Exclusion criteria

Participants with history of knee trauma, surgery, inflammatory knee pain, or history of intra-articular injections within the last 3 months and other medical disease that may affect their ability to perform exercise (e.g., uncontrolled cardiovascular, respiratory, or neurological diseases) were excluded. Patients with psychiatric diseases and communication disorder, e.g., hearing or cognitive disorders, were also excluded.

#### Data collection and tools

Demographic data of each participant about age, sex, marital status, employment status, education level and socioeconomic status [10], height, and weight were collected via a self-administered questionnaire.

The clinical variables included body mass index (BMI) [11], disease duration, and presence of comorbidities. Laboratory tests were performed such as complete blood cell picture, acute phase reactants, and serum uric acid to rule out other cause of knee pain.

Routine weight-bearing plain X-ray radiography of anteroposterior and lateral knee view was taken for the most symptomatic side. Severity was assessed, and the patients were graded according to the Kellgren–Lawrence grading system [9]. Symptomatic knee osteoarthritis was defined as the concurrent existence of frequent knee pain and radiographic findings (Kellgren–Lawrence ≥ grade 2) in the same knee [12, 13].

### Randomization

Eligible participants that met the inclusion criteria were randomly assigned to either the HEP group or the control group by a computer-generated random sequence list performed by a statistician who is independent of this study.

### Blinding

Outcome measure assessments were performed by one investigator, who was blinded to group assignment and not involved in intervention management.

### Interventions

During the first visit to the exercise and control groups, research team clarified the goals and steps of the study and presented detailed information on the etiology of osteoarthritis, how to cope with osteoarthritis, lifestyle recommendations, treatments according to guidelines, and strategies to reduce pain and enhance functional activity [14]. All participants in both groups were instructed to follow the usual care (medical treatment and lifestyle recommendations) offered by their healthcare providers throughout the study period. However, analgesics should be avoided for 24 h before follow-up assessment.

Patients randomized to the intervention exercise group were subjected to 1-h educational session on the importance of HEP; the participants were taught the exercises and conducted in front of the physiatrist to address any questions. They were given printout of illustrated booklet and videos in Arabic for exercise prescription and received a monthly phone call for motivation and to support exercise compliance. This was supported by an individual session, which took place after 3 months. During this session, the physiatrist received the exercise log to assess compliance over the previous period and focused on the beneficial role of exercise therapy and encouraging patient to continue HEP, while patients allocated to the control group did not receive HEP-related information and they were not eligible for telephone calls. Patients were then re-invited to assess any long-term progress after 6 months from the date of their initial visit.

### Home-based exercise intervention

Exercises were taught to the participants of the exercise group, as described in a previous study [15]. The HEP program included active knee joint range of motion exercises, 10 repetitions, twice per day. Stretching for the hamstring muscles was performed in a supine position with a towel wrapped around the foot, and the leg was completely extended at the knee and then raised from the position of rest until the stretch is felt behind the knee. Stretching of the quadriceps muscle was performed in sitting or prone position, with the knee flexed as much as possible; flexion was assisted by hand. The stretching was maintained for about 30 s and was carried out for 5 repetition in two sets [16]. Isometric quadriceps exercises were carried out as 10 repetitions for two sets; the patient should lie relaxed in recumbent position, and knee at 20° of flexion was supported with a rolled up towel beneath the knee and tried to press the towel to floor for 10 s. Isometric hamstring exercises were performed with knees at full extension and a towel placed under ankles by applying maximum pushing of 10 s and relaxation of 10 s, as 10 repetitions for two sets [15, 17]. They were instructed to perform the suggested exercises, three times per week for 26 weeks.

### Adherence

Adherence to HEP has been assessed via self-completed exercise log. Adherence level is calculated as the mean number of days on which patient performed the prescribed exercises during the entire intervention period. At 26 weeks, participants who completed ≥ 70% of their prescribed exercise plan were graded as high adherence, while participants with < 70% were rated as low adherence [18].

### Outcome measures

The primary outcomes were knee pain severity and health-related quality of life while secondary outcomes included exercise adherence and questionnaires to measure exercise self-efficacy.

The baseline assessments and follow-up assessments included the following:

1. The Exercise Self-efficacy Scale: the scale was developed to measure the confidence of participants in their ability to exercise regularly. “A validated Arabic version containing 18 questions, which assess how the participants were able to perform routine exercise regularly (three times per week), under certain barriers. Then, patients rated their answers using a 10-point scale ranging from 0% (I cannot do this activity at all) to 100 (I am certain that I can do this activity successfully)”. Total score was calculated by adding the numerical ratings for each item divided by the number of responses. So, higher scores indicate better self-efficacy [19].
2. Visual analog scale: the patients were asked to mark a point on a 100-mm line that represented the severity of their current pain [20].
3. Health-related quality of life: the Arabic version of 36-Item Short-Form Health Survey was used which includes eight subscale scores including the physical function, role-physical, bodily pain, energy, health perception, social function, role-emotional, and mental health, and contains 36 items. It is calculated by scoring each item on a linear scale ranging from 0 to 100, and then, all items in the same scale were averaged together. The total score ranges from 0 to 100, and higher scores reflect better health status and less disability, where 0 indicates the worst possible health status [21, 22].

### Sample size

Participation by 174 patients per group (348 total) would provide 80% power at the 5% significance level to detect difference of 7.83 with a standard deviation of 1.9 between groups in the SF-36 score [23] and an effect size of 0.31 using a two-sided t-test with alpha = 0.05 according to relevant study on exercise therapy among knee osteoarthritis patients [24]. Anticipating a 20% drop-out rate, 217 participants should be enrolled for each group.

### Statistical analysis

All data were collected, tabulated, and statistically analyzed using SPSS 23.0 for windows (IBM SPSS Statistics for Windows, Version 23.0. Armonk, NY: IBM Corp2015). Quantitative data were expressed as the mean ± SD and median (range), and qualitative data were expressed as absolute frequencies (number) and relative frequencies (percentage). Independent t-test was used to compare between two groups normally distributed. Mann-Whitney test was used to compare between two groups of non-normally distributed. Paired t test was used to compare outcome variable changes of participants in the exercise and control groups. Percent of categorical variables were compared using Chi-square test or Fisher’s exact test. Pearson’s correlation coefficient was calculated to assess relationship between various study variables, (+) sign indicates direct correlation, and (−) sign indicates inverse correlation; also, values near to 1 indicate strong correlation, and values near 0 indicate weak correlation. All tests were two sided. *P*-value ≤ 0.05 was considered statistically significant, and *p*-value > 0.05 was considered statistically insignificant.

$$ \%\kern0.5em Difference=\frac{\left|{E}_1-{E}_2\right|}{\frac{1}{2}\left({E}_1+{E}_2\right)}\ast 100 $$

where

• E_1_ is the first experimental measurement.

• E_2_ is the second experimental measurement.

## Results

**Fig. 1:**
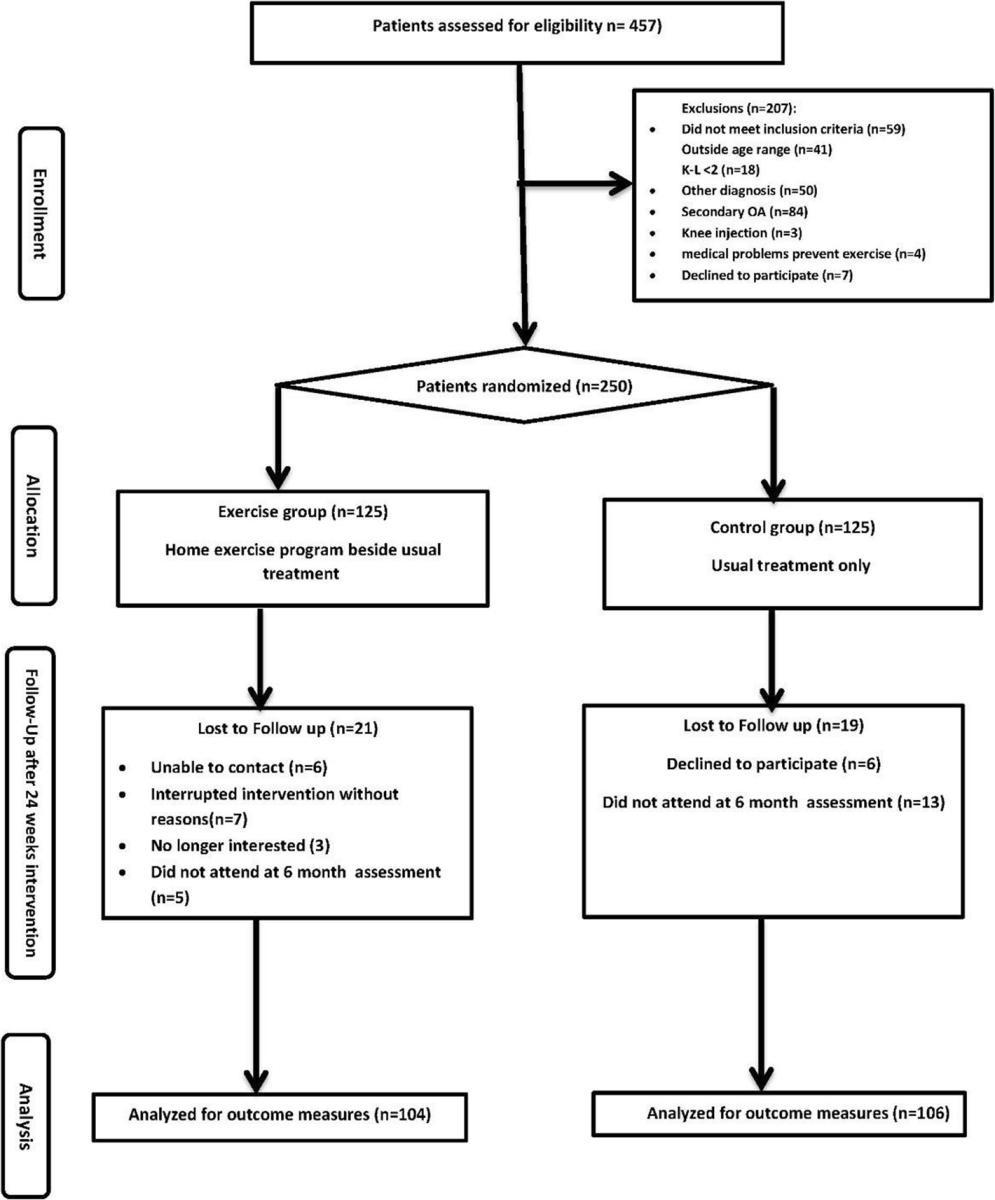
Flowchart of the study

As shown in Fig. 1, a total of 457 were screened, and 207 individuals were excluded. Among those who completed the screening, 250 participants fulfilled the eligibility criteria and were randomly allocated to either groups. Two hundred ten individuals completed the 6-month follow-up assessment (104 exercise, 83.2%; and 106 control, 84.8%).

The characteristics of the participants are described in Table 1. No significant differences were observed for demographic and clinical characteristics or any of participants’ scores of pain severity, quality of life scale, self-efficacy, and radiological severity (Kellgren-Lawrence grade) between the two groups at baseline evaluation.

**Table 1:** Demographic, clinical, and radiological characteristics of the studied groups

	Exercise group (***n*** = 104)		Control group (***n*** = 106)		Test	***p***-value
**Age (years)**						
**Mean±SD** **Median(range)**	54.9 ±7.22 55 (41–65)		55.6±6.37 55 (45–65)		t=0.74	0.45
**BMI (kg/m2)**						
**Mean± SD** **Median(range)**	27.22±4.06 25.7 (23–42.9)		27.05±3.2 26.5 (22.4–37.5)		t=0.34	0.73
**Disease duration (years)**						
**Mean± SD** **Median(range)**	6.5±3.1 6 (2–15)		5.9±2.7 5 (1–14)		t=1.49	0. 14
	No.	%	No.	%		
**Gender**						
**Male**	22	21.2	27	25.5	0.42	0.52
**Females**	82	78.8	79	74.5		
**Educational status**						
**Illiterate**	11	10.6	22	20.8	2.9	0.09
**Educated**	93	89.4	84	79.2		
**Employment**						
**Employer**	39	37.5	33	31.1		0.302
**Unemployed**	47	45.2	46	43.4	2.39	
**Manual worker**	18	17.3	27	25.5		
**Marital status**						
**Married**	62	59.6	69	65.1	0.99	0.31
**Single**	42	40.4	37	34.9		
**Residence**						
**Rural**	60	57.7	59	55.7	0.17	0.67
**Urban**	44	42.3	47	44.3		
**Smoking**						
**No**	89	85.6	87	82.1	0.32	0.56
**Yes**	15	14.4	19	17.9		
**Socioeconomic status**						
**Low**	30	28.8	24	22.6		
**Moderate**	65	62.5	75	71.7	1.84	0.39
**High**	9	8.7	7	6.6		
**Comorbidities**						
**Yes**	45	43.3	54	50.9	1.55	0.21
**No**	59	56.7	52	49.1		
**K-L grade**						
**Grade 2**	53	50.9	58	54.7		
**Grade 3**	40	38.5	41	38.7	1.13	0.57
**Grade 4**	11	10.6	7	6.6		

*χ*^*2*^ chi-square test, *t* t test, *SD* standard deviation, *BMI* body mass index, *K-L grade* Kellgren–Lawrence radiological grading

Insignificant = *p* > 0.05

Individuals who received HEP intervention experienced improvement in VAS (percent of change = −28.9%), SF36 (percent of change = 20.13%), and ESE-A (percent of change = 22.6%) at baseline compared to follow-up assessment. By contrast, those who received education showed no improvement in any of the previous outcome measures at the follow-up assessment as shown in Table 2.

**Table 2:** Outcome measures of the exercise group and the control group at baseline and 6 months post-intervention

		Studied groups		t-test	***p***	Mean difference (% of difference)
		Exercise group (***n*** = 104)	Control group (***n*** = 106)			
**VAS**	**Baseline**					
	Mean± SD Median(range)	58.29±17.4 60 (20–90)	61.2±13.7 60 (20–90)	**1.3**	**0.17**	**2.9(4.86%)**
	**After 6 months**					
	Mean± SD Median(range)	41.4±14.2 40 (20–70)	60.9±14.7 60 (20–90)	**9.7**	≤0.001*	**19.5 (38.12%)**
	*p	≤0.001*	**0.88**			
	**Mean change**	**−16.89**	**−0.3**			
	**% of change**	**−28.9 %**	**−0.49%**			
**SF 36**	**Baseline**					
	Mean± SD Median(range)	60.1±7.5 62 (43–77)	60.35±10.7 62 (35–81)	**0.15**	**0. 87**	**0.25 (0.41%)**
	**After 6 months**					
	Mean± SD Median(range)	72.2 ±10.2 73(50–92)	60.92±10.54 61 (35–81)	**7.8**	≤0.001*	**11.28 (16.9%)**
	*p	≤0.001 *	**0.074**			
	**Mean change**	**12.1**	**0.57**			
	**% of change**	**20. 13%**	**0.94%**			
**ESE_A**	**Baseline**					
	Mean± SD Median (range)	58.29± 6.9 59.4 (37.7–74.4)	55.98±11.3 55.3 (37.5–82.6)	**1.7**	**0.075**	**2.3 (4.1%)**
	**After 6 months**					
	Mean± SD Median(range)	71.5 ±7.6 72.4 (54.8–84.6)	57.72±11.1 55.9 (38.6–84.5)	**10.4**	≤0.001 *	**13.7 (21.3%)**
	*p	≤0.001 *	**0.23**			
	**Mean change**	**13.2**	**1.74**			
	**% of change**	**22.6%**	**3.1%**			

*t* t test of significance, *SD* standard deviation, *VAS* visual analog scale, *ESE-A* Exercise Self-efficacy Scale, *SF-36* Short-Form Health Survey

*Paired t test, *p ≤0.05, significant

Regarding the home exercise program adherence in exercise group, 86 (82.7%) of the patients were highly adherent to HEP, while 18 (17.3 %) patients were of low adherence level, and there were statistically significant differences (*p*≤ 0.05) between high-adherence and low-adherence groups as regards age and educational and socioeconomic status. However, no statistically significant differences (*p* > 0.05) between the two groups regarding BMI, gender, marital status, residence, and employment status were detected (Table 3).

**Table 3:** Relation of adherence level to HEP among exercise group with regard to demographic characteristics

	Exercise group				test	***p***-value
	High adherence ***n*** = 86		Low adherence ***n*** = 18			
	No.	%	No.	%		
**Age (years)**						
Mean± SD Median (range)	54.9 ±7.1 54.5 (41–65)		59.2 ±5.8 59 (50–65)		t = 2.4	0.015*
**BMI (kg/m**^**2**^**)**						
Mean± SD Median(range)	27.15±4.3 25.3 (23–43)		27.6±2.8 26.5 (23–33)		t = 0.41	0.68
**Gender**						
Male (22)	20	90.9	2	9.1	f	0.56
Females (82)	66	80.49	16	19.51		
**Education**						
Illiterate (11)	6	54.5	5	45.5	9.3	0.002*
Educated (93)	80	86.1	13	13.9		
**Employment**						
Employer (39)	33	84.62	6	15.38		
Unemployed (47)	39	82.9	8	17.1	0.41	0.81
Manual worker (18)	14	77.78	4	22.22		
**Marital status**						
Married (62)	53	85.48	9	14.52	1.3	0.25
Single (42)	33	78.6	9	21.4		
**Residence**						
Rural (60)	52	86.7	8	13.3	1.1	0.29
Urban (44)	34	77.27	10	22.73		
**Smoking**						
No (89)	71	79.8	18	20.2	F	0.067
Yes (15)	15	100.00	0	.00		
**Socioeconomic status**						
Low (30)	19	63.33	11	36.67	10.5	0.005*
Moderate (65)	58	89.2	7	10.8		
High (9)	9	100.00	0	.00		

*χ*^*2*^ chi-square test, *t* t test, *f* Fisher exact test, *SD* standard deviation, *BMI* body mass index

*p≤0.05, significant

As regarding clinical manifestations, there was no statistically significant differences (*p* > 0.05) between adherent and non-adherent groups except for patients with comorbidities (*p*≤ 0.05) where absence of other comorbidities was significantly associated with high adherence level for HEP. Also, there was statistically significant improvement within high-adherence group in all evaluated outcome scores after 6 months (Table 4).

**Table 4:** Relation of adherence level to HEP among exercise group with regard to clinical characteristics and outcome measures

	Exercise group				test	*p*-value
	High adherence ***n*** = 86		Low adherence ***n*** = 18			
	No.	%	No.	%		
**Disease duration (years)**						
Mean± SD Median (range)	6.57±3.5 6 (2–15)		7.5±2.7 6 (4–14)		t=1.1	0.26
**Comorbidities**						
Yes (45)	31	68.89	14	31.11	9	0.003*
No (59)	55	93.2	4	6.8		
**K-L grade:**						
Two (53)	42	79.25	11	20.75	0.88	0.64
Three (40)	34	85	6	15		
Four (11)	10	90.91	1	9.09		
**VAS (baseline)**						
Mean± SD Median(range)	56.9±18.3 60 (20–90)		64.2±11.2 60 (30–80)		MW=1.7	0.08
**SF-36 (baseline)**						
Mean± SD Median (range)	61.1±7.5 63 (43–77)		57.9±5.8 58 (45–71)		t=1.7	0.08
**ESE A (baseline)**						
Mean± SD Median(range)	58.6±7.3 59.4 (37.7–74.4)		56.3±4.6 56.6 (48.3–65)		t=1.3	0.19
**VAS (6month)**						
Mean± SD Median (range)	38.7±13.4 40 (20–60)		50.6±9.8 50 (30–70)		t=2.1	0.0004*
**SF-36 (6month)**						
Mean± SD Median (range)	74.1±9.7 73 (52–92)		64.1±10.7 61 (50–86)		t=3.9	≤0.001*
**ESE A (6month)**						
Mean± SD Median (range)	72.8 ±9.8 73.2 (54.8–84.6)		61.6 ±4.6 60 (55.9–68.3)		MW=5.8	≤0.001*

*χ*^*2*^ chi-square test, *t* t test, *MW* Mann-Whitney test, *f* Fisher exact test, *K-L grade* Kellgren–Lawrence radiological grading, *SD* standard deviation, *VAS* visual analog scale, *ESE-A* exercise self-efficacy scale, *SF-36* Short-Form Health Survey

*p≤0.05, significant

**Fig. 2:**
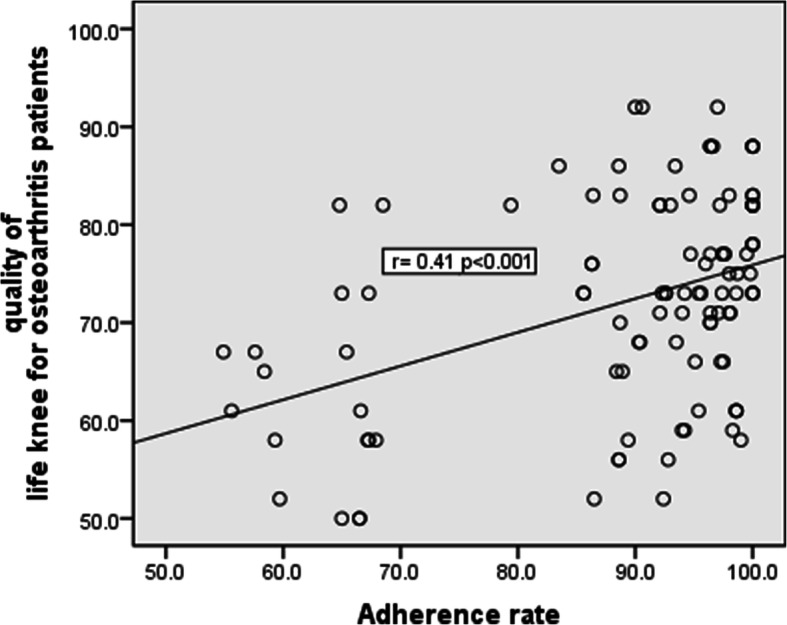
Scatter diagram for correlation between adherence rate and quality of life of osteoarthritis patients after intervention

**Fig. 3:**
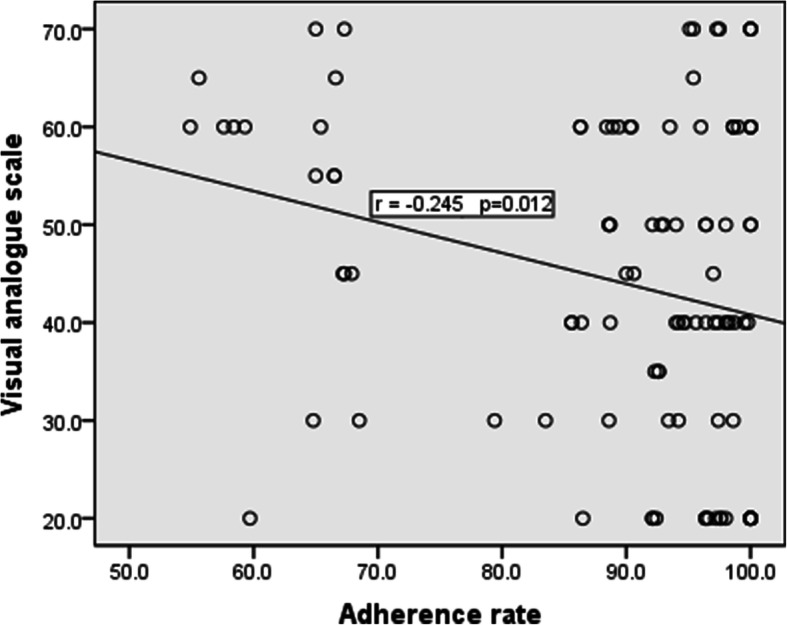
Scatter diagram for correlation between adherence rate and visual analog scale of osteoarthritis patients after intervention

Additionally, there was statistically significant negative correlation between adherence rate and age of patients per years and also with visual analog scale (*p*≤ 0.05), while there was statistically significant positive correlation between adherence rate and quality of life, also with self-efficacy after intervention at 6 months (*p*≤ 0.05) suggesting an improvement in pain severity, quality of life, and exercise self-efficacy scale-Arabic scores with high adherence levels (Table 5, Figs. 2 and 3).

**Table 5:** Correlation between home exercise adherence rate and some of the related factors and outcome measures after (6 months) home exercise period

Parameters	Adherence rate	
	(*r*)	*p*
**Age per years**	−0.231	0.018*
**Disease duration per years**	−0.181	0.064
**BMI**	−0.064	0.516
**VAS at 6 months after intervention**	−0.245	0.012*
**SF-36 at 6 months after intervention**	0.41	<0.001*
**ESE_A at 6 months after intervention**	0.73	<0.001*

*(r)* correlation coefficient, *BMI* body mass index, *VAS* visual analog scale, *ESE-A* Exercise Self-efficacy Scale, *SF-36* Short-Form Health Survey

*p≤0.05, significant

## Discussion

To the best of authors’ knowledge, this is the first study in Egypt to evaluate the effect of home-based exercise program intervention on self-efficacy and quality of life in primary KOA patients in a controlled manner.

The present results indicated that the addition of 26 weeks of HEP with the usual care resulted in improvement of self-efficacy, pain severity, and health-related quality of life among symptomatic primary KOA patients. Also, the adherence to HEP may have an impact on patient improvement.

The present result agreed with a previous randomized controlled study of 786 participants with knee pain that improvements in pain, stiffness, and physical function were found after 6 months of exercise program [25].

Previous studies indicated that quadriceps muscle simple home exercise programs could significantly improve self-reported knee pain and disability among knee osteoarthritis patients as well as the results for quality of life [26]. Another Jordanian research found that participants who exercised more often and for longer periods had more exercise self-efficacy [19].

The improvement in pain could be attributed to the increased discharges from mechanosensitive afferent nerve fibers A-delta and IV (C) resulting from skeletal muscles secondary to rhythmic muscle contraction that can stimulate central opioid systems, resulting in pain relief and a reduction in disability. Exercise has also been shown to improve blood beta-endorphin levels [27].

Since the efficacy of any therapeutic exercise program is directly linked to patient compliance [28, 29], measures to increase efficiency by optimizing adherence should be taken when setting up exercise programs for patients with knee osteoarthritis [30].

So, the adherence level in the exercise group of patients was examined, and it was noticed that 86 (82.7%) of the patients were highly adherent to the home exercise program while 18 (17.3 %) patients were of low adherence level. In agreement with the findings of the previous reports, the frequency of completely adherent patients to their prescribed HEP is approximately 53–89% [31].

It is important to note that the adherence rate in another study was higher with written and verbal information (77%) than those given verbal information only (38%), which is consistent with our finding [32]. The high rate of adherence recorded in this study was also enhanced by monthly telephone support to the enrolled patients and direct physician access after 3 months. The authors presume that the wide range reported in other studies may be a consequence of varying sample sizes, different patient characteristics, different intervention methods, and the inclusion of different outcome measures.

In addition, several factors affecting adherence have been identified in this study. There was a statistically significant difference between high-adherence and low-adherence groups in terms of age, educational level, socioeconomic status, and the presence of other co-morbidities (*p* ≤ 0.05). However, no statistically significant differences (*p* > 0.05) between the two groups were identified with regards to BMI, gender, marital status, residence, employment status, disease duration, and radiological grades.

Similarly, another study determined the relationship between adherence to HEP and age [33, 34]. Also, it has been previously reported that educated patients were more adherent than uneducated ones as educated patients usually have better health literacy and would know the importance of adhering to treatment [35].

As far as comorbidity and exercise compliance is concerned, the present result agreed with a previous descriptive cross-sectional study that patients with chronic diseases continue to report exercises of less than the optimum amount despite the need to exercise [36].

Most notably, the clinical condition of patients with KOA during the COVID-19 surge can be enhanced if patients are contacted regularly by phone or engaged in live virtual sessions with their physician [4]. As a result, remote treatment may be a more effective and cost-effective solution for preventing overburdened health institutions and reducing healthcare spending or face-to-face visits [37].

Furthermore, HEP can be recommended as an effective strategy for knee osteoarthritis patients that may be implicated for clinical practice. Further future research with a large population sample and for long term is recommended to confirm these findings.

### Study limitations

One of the limitations of the current study is related to the subjective assessment of adherence level as it was subjective being based on a personal documentation. In addition, this study is considered a short-term intervention that did not allow for evaluation of long-term effects of HEP.

Also, the HEP did not include static or dynamic balance exercises for postural control, neither muscle endurance exercises which must be addressed in further studies.

## Conclusion

Home-based exercise program improves pain score, self-efficacy, and quality of life in patients with knee osteoarthritis. Adherence level to the exercise program may have a positive impact on patient improvement.

## Supplementary Information

**Additional file 1.** Knee osteoarthritis guide.

## Data Availability

The data will be available upon request.

## Abbreviations

ACR: American College of Rheumatology
BMI: Body mass index
COVID-19: Coronavirus disease 2019
ESE-A: Exercise Self-efficacy Scale-Arabic
HEP: Home exercise programs
HRQOL: Health-related quality of life
K-L: Kellgren–Lawrence grading
KOA: Knee osteoarthritis
ROM: Range of motion
SF-36: 36-Item Short-Form Health Survey
VAS: Visual analog scale
