# Supplementary material for: Impact of home exercise program on self-efficacy and quality of life among primary knee osteoarthritis patients: a randomized controlled clinical study
Source: Egypt Rheumatol Rehabil. 2021 Jun 24;48(1):28. doi: 10.1186/s43166-021-00073-2 (PMC8223192; doi:10.1186/s43166-021-00073-2)

**التعليمات والنصائح لمرضي خشونة الركبة:**

هذا الكتيب يعرض بعض المعلومات والنصائح الخاصة بمرض خشونة الركبة و وصف لبعض التمارين التي يمكن ان تساعد على تخفيف حدة الالم وزيادة الكفاءة الوظيفية كما يتيح الرد على بعض الأسئلة الشائعة عن هذا المرض.

- **ما هو مرض خشونة الركبة؟**

هو حالة مرضية يصاب فيها الشخص بعدم قدرة الغضاريف علي توفير الدعامة المرنة بين عظام المفصل و من ثم تتكون النتوءات العظمية و مع الوقت يحدث تلامس لعظام مفصل الركبة مع الحركة، وذلك نتيجة ترقق الفاصل الغضروفي. كذلك قد يتسبب هذا الاحتكاك في حدوث تورم بالمفصل وقصور في الحركة

**ما هي اسباب مرض خشونة الركبة؟**تتعدد أسباب خشونة المفاصل المبكرة بشكل عام وبالتحديد خشونة الركبة ومن اهمها الوضع الخاطئ في الجلوس لفترات طويلة، كثني الركبة أثناء الجلوس. - سوء التغذية وعدم تناول الأطعمة التي تحتوي على الكالسيوم وكذلك فيتامين د وفيتامين سي. - زيادة الوزن أيضاً تزيد من التحميل على مفصل الركبة مما يسبب تآكل الغضاريف. هذا بالإضافة الى وجود عوامل وراثية لخشونة الركبة و قلة مرونة الانسجة الرخوة والقصور في مجال حركة المفاصل الناتج عن عدم اللياقة البدنية.

- **ما هي اعراض خشونة الركبة؟**يشعر المصاب بأعراض مختلفة وأبرزها آلام في الركبة مصحوبه بسماع صوت طقطقة في الركبة عند الحركة و الشعور بألم شديد عند صعود السلم و عدم القدرة على الجلوس بوضع القرفصاء والشعور بألم شديد حينها كما قد يحدث تورم بالركبة.
- **ما هو علاج خشونة الركبة؟**
- كل ما تستطيع فعله هو وقف تدهور الحالة والحياة بشكل طبيعي طوال حياتك.
- مضادات الالتهاب الخالية من الستيرويدات لتخفيف الالام الحادة والمتوسطة
- [المسكنات الموضعية:](https://www.webteb.com/articles/10-%D8%A7%D8%AE%D8%B7%D8%A7%D8%A1-%D8%B4%D8%A7%D8%A6%D8%B9%D8%A9-%D9%84%D8%AF%D9%89-%D8%A7%D8%B3%D8%AA%D8%B9%D9%85%D8%A7%D9%84-%D9%85%D8%B3%D9%83%D9%86%D8%A7%D8%AA-%D8%A7%D9%84%D8%A7%D9%84%D9%85_1764) وهي كريمات ومراهم طبية من الممكن دهنها على الركبة المصابة لتخفيف الألم والتورم.
- الحقن: هناك نوعان من الحقن المتوفر لعلاج خشونة الركبة وهما حقن الستيرويدات وحقن حمض الهيالورونيك.
- مكملات غذائية كالجلوكوزامين وسلفات الكوندرويتين: وهي مواد ينتجها عادة الجسم بشكل طبيعي في داخل الغضاريف نفسها ويمكن إعطائها لدعم غضاريف الركبة وتقويتها.

**ما هي اهم النصائح لمرضى خشونة الركبة؟**

- الاهتمام بصحة الركبة والحفاظ عليها يجنّبك الإصابة بأمراض العظام المزمنة مع التقدم في العمر.
- تقليل الوزن الزائد هي أهم خطوة في [علاج خشونة الركبة](https://hip-knee.com/%D8%AE%D8%B4%D9%88%D9%86%D8%A9-%D8%A7%D9%84%D8%B1%D9%83%D8%A8%D8%A9/#treat).
- الحرص على تناول الأطعمة المفيدة الغنية ب[الكالسيوم](https://www.elconsolto.com/medical-advice/Tag/7677/%D8%A7%D9%84%D9%83%D8%A7%D9%84%D8%B3%D9%8A%D9%88%D9%85#bodykeywords)والمغنسيوم المواظبة على التعرض المباشر لأشعة الشمس، للحصول على[فيتامين د](https://www.elconsolto.com/medical-advice/Tag/6887/%D9%81%D9%8A%D8%AA%D8%A7%D9%85%D9%8A%D9%86-%D8%AF#bodykeywords)اللازم لصحة العظام و الغضاريف. وكذلك شرب المياه والذي يساعد على التخفيف من الام الركبة.
- عمل تمارين استطالة لعضلات الفخذ الامامية والخلفية والسمانة ثلاثة مرات أسبوعيا
- المواظبة على ممارسة التمارين الرياضية اللازمة لتقوية عضلات الفخذ الأمامي والخلفي بأوزان خفيفة ومتوسطة.
  و يمكنك وضع كمادات ثلج بعد التمرين.

**إذا كنت تعاني من خشونة بالركبة فعليك بالآتي:**

- تجنب الوقوف لفترات طويلة
- تجنب ثني الركبتين أثناء الجلوس.
- الحرص على القيام بتمرينات الاستطالة قبل ممارسة التمارين الرياضية.
- إذا كنت ترغب في ممارسة رياضة ركوب الدراجة فيمكنك استخدام الدراجة المزودة بكرسي والتبديل بالبدال والركبة مفرودة نسبياً.
- عند الصعود ضع قدمك السليمة في الأمام أولا ثم أصعد بالرجل الصحيحة أو الأقل ألما الى نفس درجة السلم و ليس التالية لها. و العكس عند النزول  حيث عليك بالبدء بالقدم المريضة أولا ثم تهبط بالرجل السليمة الى نفس الدرجة

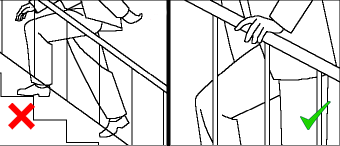


**هل المشي يؤدي الى تفاقم خشونة الركبة؟**

لا علي العكس بل ينصح بالمشي لمدة تتراوح ما بين 20 إلى 35 دقيقة يوميا، مع عدم الإفراط، وتجنب السير على الأرضيات الغير مستوية أو المائلة لأعلى أو لأسفل او صعود السلالم بكثرة ، بالإضافة إلى الحرص على ارتداء حذاء مريح ومناسب.
واما بالنسبة لكبار السن و المرضى الذين يعانون خشونة متقدمة أن يستخدموا عكاز طبى أثناء المشي على الناحية الأقل تأثرا وتحريك العصا و الرجل المصابة في آن واحد للمساعدة في التخفيف من الحمل الواقع على المفصل**.**

**تمارين اطالة عضلات الفخذ الخلفية:**

**تمرين1:**

- ولتمارين الشد إستلقى على ظهرك، لف شريط من القماش حول القدم على شكل حلقة، مع المحافظة على الساق مستقيمة، إستخدم الشريط للمساعدة على شد القدم لأعلي .
-
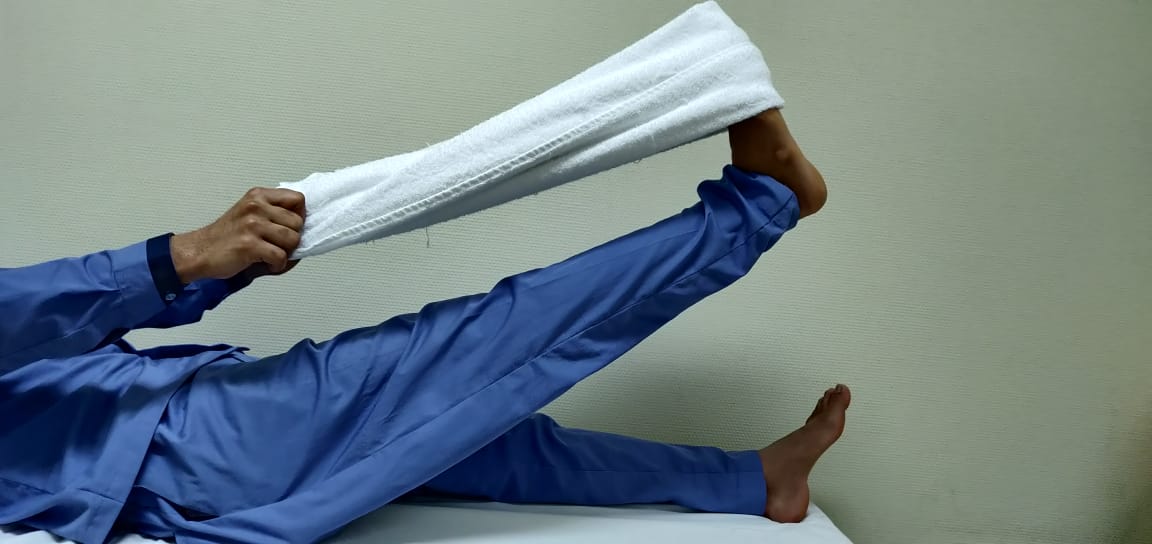
إنتظر 20 ثانية، ثم إخفض الساق ، ثم قم بعمل التمرين للقدم الأخرى و كرر التمرين خمس مرات فقط يومياً .

تمرين استاتيكية لتقوية العضلات الخلفية للفخذين:

**تمرين2:**


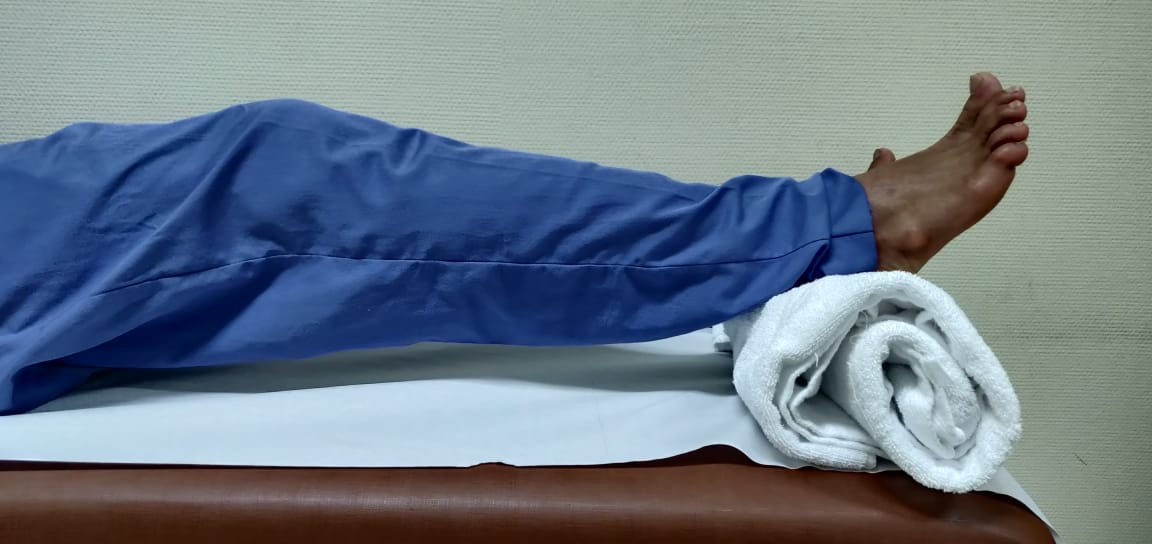


قم بالاستلقاء على الظهر مع ثنى بسيط على الركبة مستندا بالكاحل على السرير أو الأرض. اضغط بكاحلك على السرير أو الأرض للأسفل مع الحفاظ على الركبة مفرودة. من المفترض ان تشعر بانقباض في العضلات الخلفية للفخذ. انتظر لمدة20 ثانية ثم استرخ لمدة 10 ثوان اخرى قم بعمل مجموعتين من 10 عدات مع تكرار التمرين للساق الأخري.

**تقوية عضلات الفخذ الامامية:**

**تمرين3:**


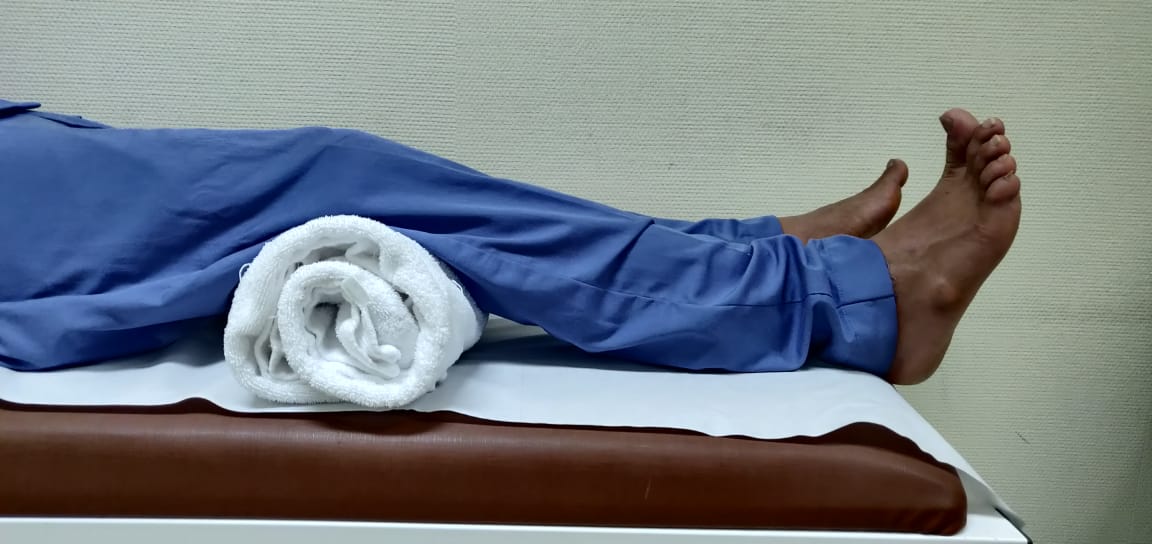


استلق على ظهرك ، ضع منشفة صغيرة أسفل الركبة. اضغط بركبتك على المنشفة ستشعر بشدّ وانقباض عضلة فخذك الأمامية وكذلك سترى الردفة (العظم المتحرك في رأس الركبة) ترتفع للأعلى باتجاه الفخذ وهي دلالة على إتمام انقباض العضلة.  احتفظ بهذا الوضع مدة 10 ثوانٍ ثم استرح تدريجيا وكرر التمرين**. قم** بعمل مجموعتين من 10 عدات مع التبديل بالساق الاخرى.

**تمرين 4:**  **
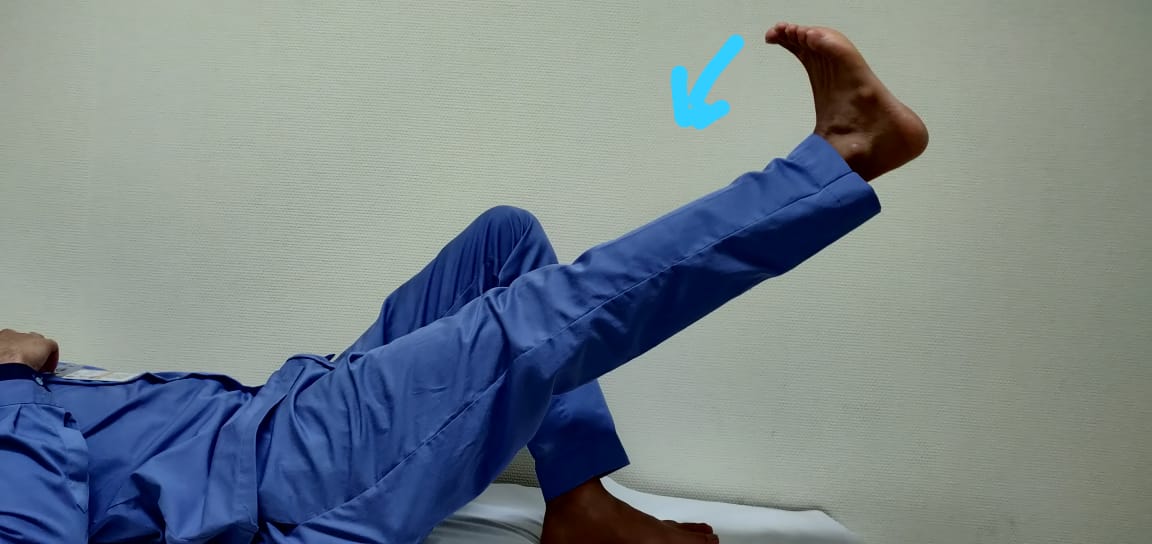
**

إستلقى على ظهرك، إرفع جسمك بمساعدة مرفقيك، قم بثنى ركبتك اليسرى، وقدمك على الأرض مع الحفاظ على ساقك اليمنى مستقيمة، وأصابع قدميك لأعلى، شد عضلات الفخذ، ثم أرفع ساقك اليمنى لأعلى. مع الحفاظ علي شد القدم لأعلي إنتظر 10 ثوانى على هذا الوضع، وحافظ على عضلات الفخذ مشدودة ، وببطء إخفض ساقك على الأرض، إلمسها ثم أعد رفع ساقك مرة أخرى بنفس الطريقة. بدل للساق الأخرى وطبق عليها نفس التمرين، وقم بعمل مجموعتين من 10 عدات.

**تمرين 5**:


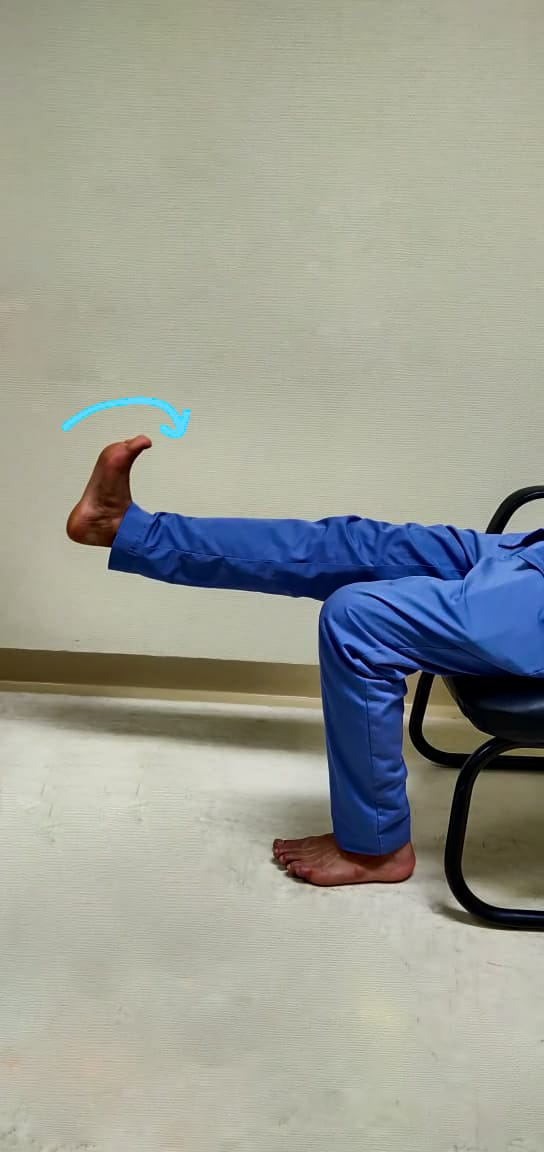


يتم الجلوس على الكرسي في وضع مستقيم ويتم مد الساق اليمنى إلى الأمام مع رفعها مستقيمة إلى مستوى الركبة الأخرى كما يجب أن يكون الكاحل مثنيا نحو الركبة وأن تكون أصابع القدم نحو الاعلى والبقاء على هذه الوضعية مدة 10 ثوان، ثم الهبوط بالساق على الأرض ببطء ويكرر التمرين بالساقين مع رفع وخفض الساق 10مرات.

تمرين اطالة عضلات الفخذ الامامية:

**تمرين** 6:

يتم الوقوف والاستناد على ظهر كرسي أو حائط للدعم قم بلف شريط من القماش حول الكاحل الأيسر على شكل حلقة مع ثنى الركبة للخلف قدر المستطاع و الشد على الكاحل في نفس الاتجاه. إنتظر 20 ثانية ثم إخفض الساق. كرر التمرين 5 عدات ، ثم بدل للقدم الأخرى وطبق عليها نفس التمرين ونفس الإعادات.


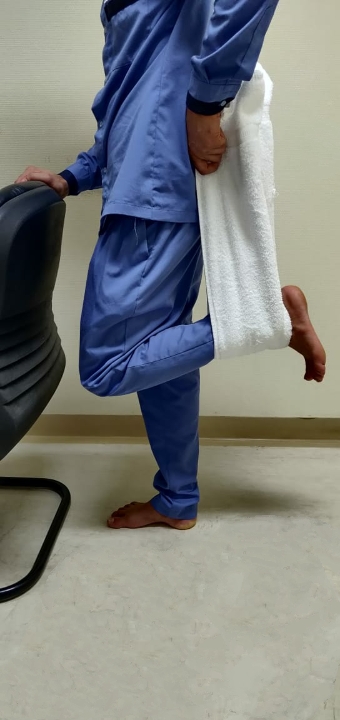


**تمرين 7 (رفع الكعب):**

يتم الوقوف والاستناد على ظهر كرسي أو حائط للدعم مع ورفع الكعب عن الأرض والوقوف على أصابع القدم ، حافظ على هذا الوضع لمدة 10ثوانى، وببطء اخفض كعبيك الإثنين إلي الأرض.قم بعمل مجموعتين من 5 مع فترات راحة قصيرة بينهما.


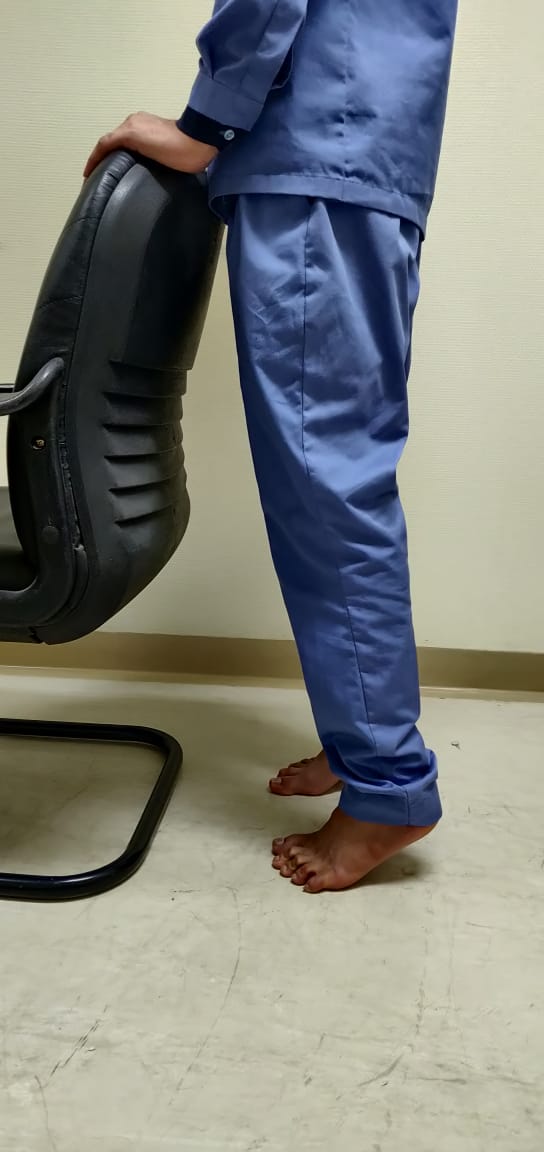

Supplement: Supplementary file 1 — Additional file 1. Knee osteoarthritis guide. [file 43166_2021_73_MOESM1_ESM.zip › 43166_2021_73_MOESM1_ESM/ARABIC BOOKLET FINAL (1).docx]
