# Supplementary material for: Impact of home exercise program on self-efficacy and quality of life among primary knee osteoarthritis patients: a randomized controlled clinical study
Source: Egypt Rheumatol Rehabil. 2021 Jun 24;48(1):28. doi: 10.1186/s43166-021-00073-2 (PMC8223192; doi:10.1186/s43166-021-00073-2)

**Knee osteoarthritis guide**

**Knee osteoarthritis Advice and Exercises**

This booklet provides general information about knee osteoarthritis and simple exercises that may help pain alleviation and functional improvement. It also, provides information and answers to your questions about this condition.

**What is knee osteoarthritis?**

It is a degenerative disease of the knee joint. It is typically characterized by wear and tear and gradual damage of articular cartilage which acts as a cushion between articular surfaces. Progressive loss of articular cartilage increases friction between joints resulting in joint pain, stiffness, inflammation, swelling and spurs formation.

**What are the causes of osteoarthritis?**

There are variable factors that can increase risk of osteoarthritis in general, specifically knee osteoarthritis, the most important of which is the wrong position of sitting for long periods, such as squatting. Poor nutrition and lack of foods containing calcium, vitamin D or vitamin C. Being overweight increases the loads on the knee joint, causing cartilage wear. This is in addition to the presence of genetic factors for knee osteoarthritis, decreased flexibility of soft tissues, and the range of motion of the joints resulting from neglecting stretching exercises.

**What are the symptoms of knee osteoarthritis?**

Common clinical symptoms include knee pain with crackly sound that increases with activity with little improvement with rest, knee stiffness, and decrease in knee mobility, making it difficult to go upstairs, walk or squat and may be associated with knee swelling.

**How Is Osteoarthritis of the Knee Treated?**

The primary goals of [management of knee osteoarthritis](https://www.webmd.com/osteoarthritis/osteoarthritis-treatment-options) are to stop joint deterioration, relieve symptoms, and restore function to improve your quality of life.

You can use one or more of the following drugs:

- Nonsteroidal anti-inflammatory drugs for mild and moderate pain relief.
- Topical anti-inflammatory creams and ointments are available to relieve pain and reduce swelling affecting joints
- Injection: intraarticular injection of corticosteroids or [hyaluronic acid](https://www.webmd.com/vitamins-supplements/ingredientmono-1062-hyaluronic+acid.aspx?activeingredientid=1062&activeingredientname=hyaluronic+acid) into the knee.
- Glucosamine and chondroitin sulfate supplements: These are substances that exist within the body as a natural building within the cartilage itself to support and strengthen it.

**What are the most important measures to help knee osteoarthritis management?**

- Healthy lifestyle habits like good nutrition, weight control and exercise are recommended for healthy knee joint in order to decrease osteoarthritic changes with aging.
- Weight control is the most important step in treatment of knee osteoarthritis
- In general, eating more fresh vegetables and fruits rich in calcium and magnesium, and regular exposure to direct sunlight, to obtain vitamin D, which is necessary for bone and cartilage health. Drinking water helps relieve knee pain.
- Stretching exercises for muscles supporting the knee join are encouraged.
- Regular exercises for strengthening muscles around knee joint as well as using cold pack after exercise.

**Warning**

**If you have knee osteoarthritis you should avoid /do the following:**

- Avoid standing for extended periods of time.
- Avoid frequent knee bending
- If you think about bicycling, you can use a bike that is fitted with a seat that is set at optimal heights allowing the knee to be relatively straight as you pedal.
- Performing stretching exercises before exercising.
- When climbing up stairs, lead with your good leg, and then go up with the affected leg to the same step, not the next one, and vice versa when you go downstairs, you must lead with the affected leg first and then descend with the healthy leg to the same step.


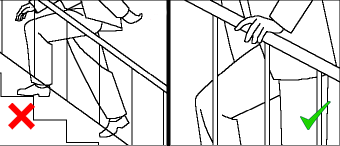


**Does walking exacerbate knee osteoarthritis?**

No, it is recommended to walk for a period of between 20 to 35 minutes a day, and to avoid walking on uneven floors or tilted up or down or climbing stairs frequently, in addition to wear comfortable and appropriate shoes. As for the elderly and patients with severe osteoarthritis, they should use a walking stick while walking and hold it the opposite side from the weak or painful leg and moving the cane with the painful limb when you step forwards to help reduce the burden on the joint.

**Knee Osteoarthritis Exercises**

# Exercise 1:

**Hamstring muscle stretch:** Lie down when you're ready to stretch your hamstring. Loop a bed sheet around your right foot. Use the sheet to help pull the straight leg up. Hold for 20 seconds, and then lower the leg. Perform the exercise for repetitions. Then, switch legs.


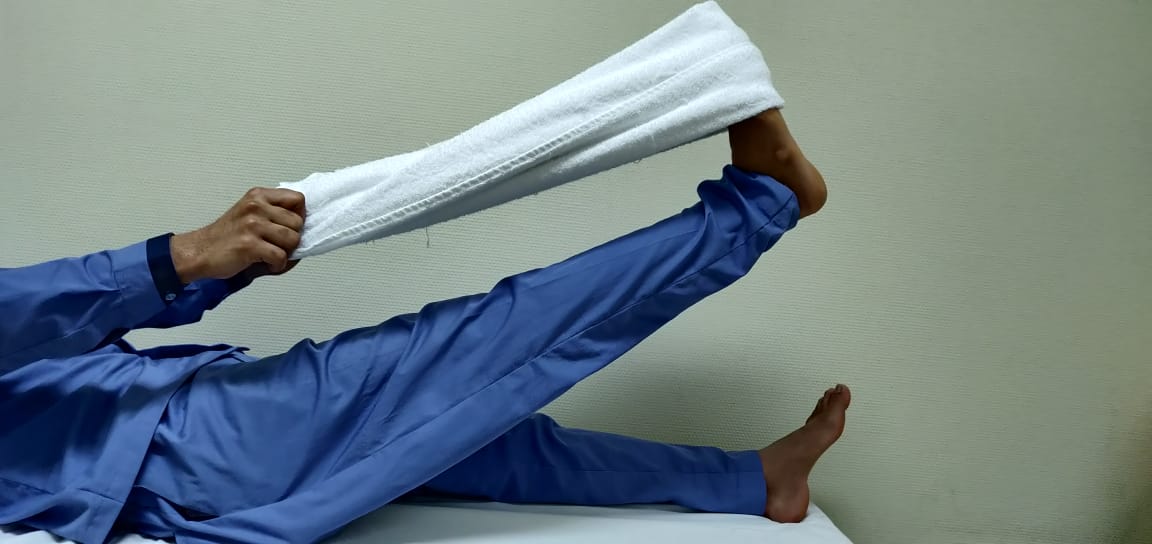


**Exercise 2:**

**Hamstring set:** Lie flat on your back, with your right knee slightly bent and your heel on the floor/mat then, press the back of your heel firmly down. You should feel the muscles in the back of your thigh tightening. Hold for 10 seconds and relax. Perform 2 sets of 10 repetitions. Switch legs after each set.


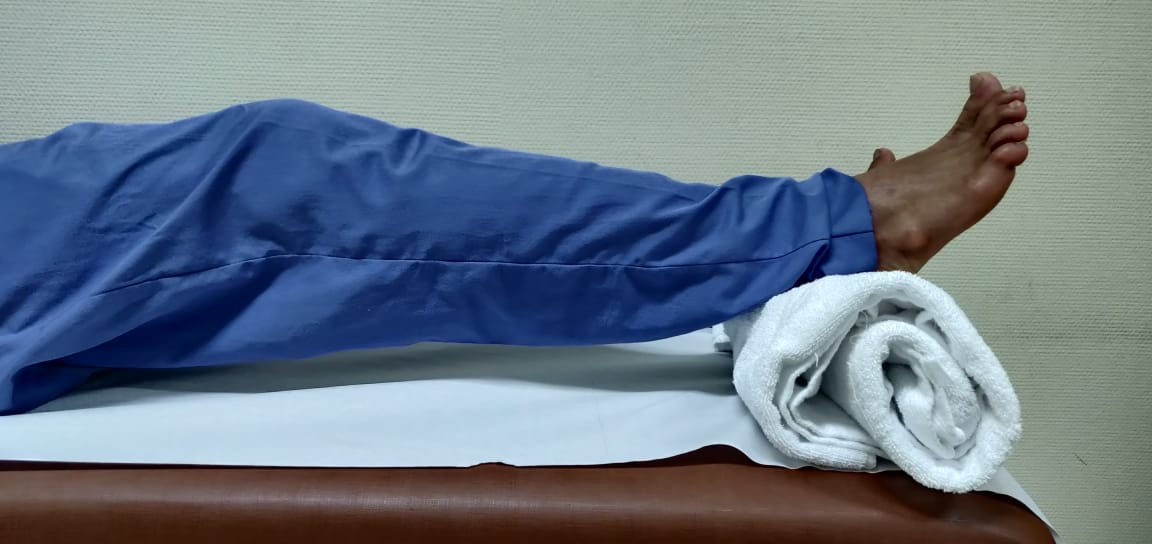


**Exercise 3:**

**Quadriceps set:** Lie on your back with the leg you want to exercise straight.  Place a small rolled towel underneath the knee. Slowly tighten the muscle on top of the thigh (quadriceps) and push the back of the knee down into the rolled towel.  Hold contraction for 10 seconds and then slowly release, resting 10 seconds between each contraction. Perform 2 sets of 10 repetitions. Switch legs after each set.


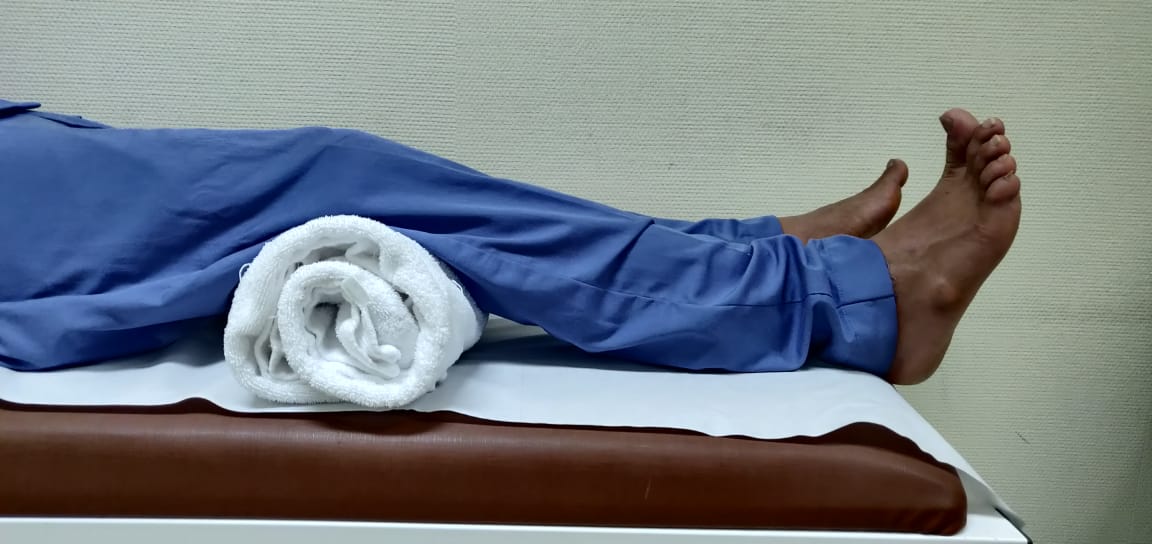


**Exercise 4:**

**Straight Leg Raise:** Lie on the floor, upper body supported by your elbows. Bend your left knee, foot on the floor. Keep the right leg straight, toes pointed up and dorsiflexion at the ankle. Tighten your thigh muscles and raise your right leg. Pause, as shown, for 10 seconds. Keep your thigh muscles tight and slowly lower your leg to the ground. Touch and raise again. Do two sets of 10 repetitions. Switch legs after each set.


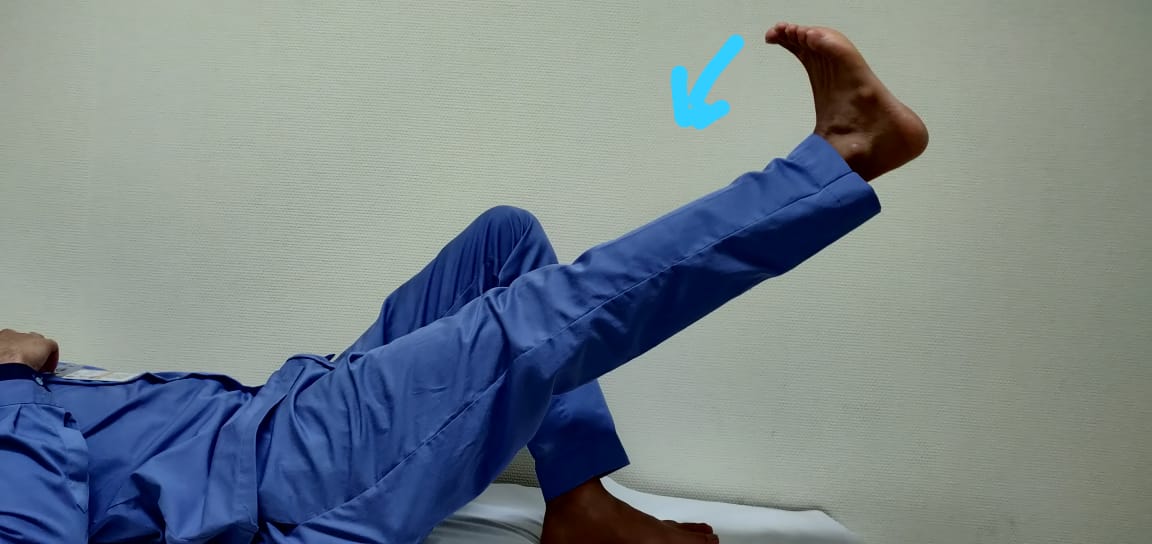


**Exercise 5:**

**Seated Leg Raise**: Sit on a chair with both legs bent at 90°. Slowly raise your right leg so that it is parallel to the floor with ankle dorsiflexion, keeping your left foot on the ground. Hold for 10 seconds, then slowly bring the right foot back to the floor, and repeat on left leg. Do one set of 10 repetitions. Then, switch legs


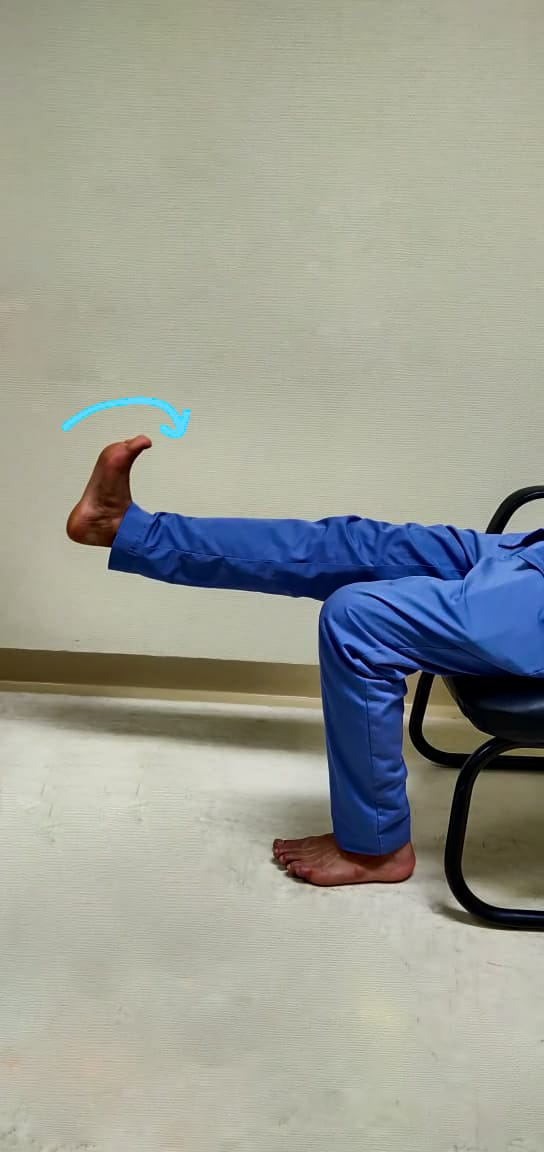


**Exercise 6:**

**Quadriceps stretching:** Stand up straight and hold onto a wall or the back of a chair for support. To perform this stretch, Loop a sheet around your left foot. Pull your left foot toward your buttock with bending your left knee as tolerable. Hold the stretch for 20 seconds, release and repeat on the right leg. Stretch each leg for five times in a stretching session.


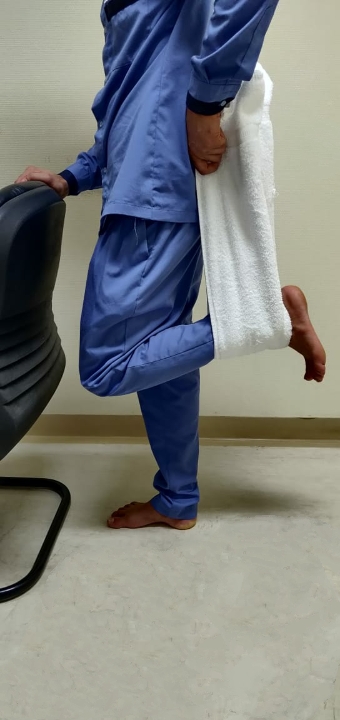


**Exercise 7:**

**Heel Raise:** Stand tall and hold the back of a chair for support. Lift your heels off the ground and rise on the toes of both feet. Hold for 10 seconds. Slowly lower both heels to the ground. Do two sets of 5 repetitions.


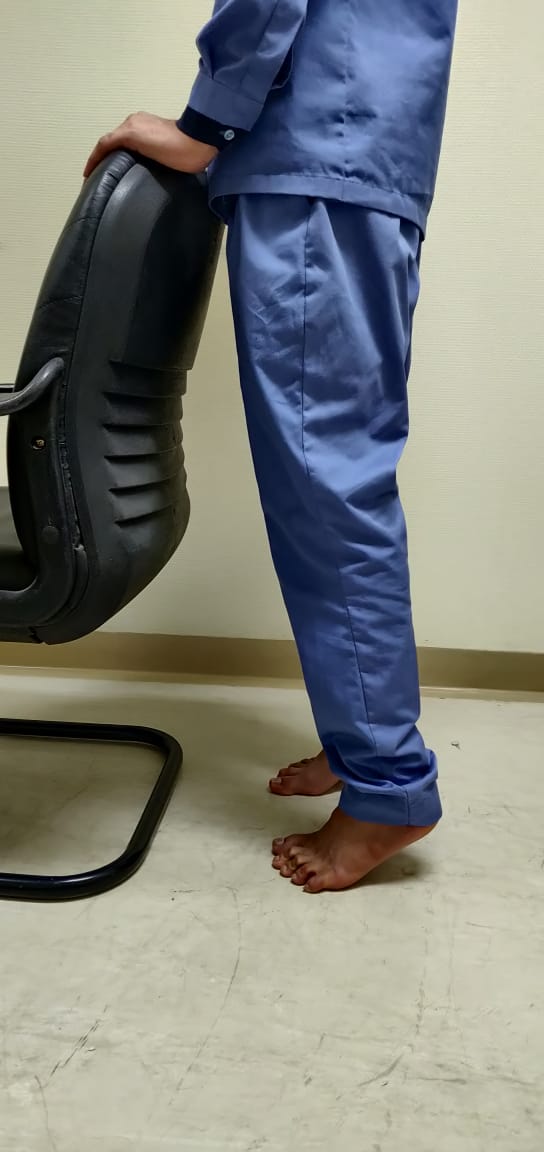

Supplement: Supplementary file 1 — Additional file 1. Knee osteoarthritis guide. [file 43166_2021_73_MOESM1_ESM.zip › 43166_2021_73_MOESM1_ESM/english booklet final version.docx]
